# Association of sonographic features and clinicopathologic factors of papillary thyroid microcarcinoma for prevalence of lymph node metastasis: a retrospective analysis

**DOI:** 10.20945/2359-3997000000297

**Published:** 2020-10-09

**Authors:** Quan Zou, Sumei Ma, Xinghu Zhou

**Affiliations:** 1 The First Hospital of Lanzhou University Department of Ultrasound Lanzhou China Department of Ultrasound, The First Hospital of Lanzhou University, Lanzhou, China; 2 The First Hospital of Lanzhou University Department of Cardiology Lanzhou China Department of Cardiology, The First Hospital of Lanzhou University, Lanzhou, China

**Keywords:** Clinicopathologic factors, lymph node metastasis, lymph node resection, papillary thyroid microcarcinoma, sonographic features, thyroidectomy

## Abstract

**Objective:**

The objective of the study was to develop an association between clinicopathologic and sonographic features of patients with papillary thyroid microcarcinoma and the prevalence of lymph node metastasis.

**Subjects and methods:**

Clinicopathologic and sonographic features of 415 patients of papillary thyroid microcarcinoma with (n = 102) or without (n = 313) lymph node metastasis were retrospectively reviewed. The thickness of the lymph node ≥ 6 mm with intra-lymph nodal occupying lesions considered lymph node metastasis. Also, it was considered metastasis if lymph node perfusion or blood flow defect was found with any thickness size. Univariate following multivariate analysis was performed for the prediction of sonographic features and clinicopathologic factors for the prevalence of lymph node metastasis.

**Results:**

Male gender ( *p* = 0.041), age < 45 years ( *p* = 0.042), preoperative calcitonin > 65 pg/ mL ( *p* = 0.039), nodule size > 5 mm in diameter ( *p* = 0.038), bilaterality ( *p* = 0.038), tumor capsular invasion ( *p* = 0.048), cystic change ( *p* = 0.047), and hyper vascularity ( *p* = 0.049) of thyroid nodules were associated with lymph node metastasis. Also, thyroid nodules 5 mm and more in diameter may have high aggressiveness.

**Conclusion:**

These data helped the surgeon for individualized treatment in thyroid carcinoma and avoid unnecessary prophylactic surgery of the lymph node.

## INTRODUCTION

Thyroid carcinoma measuring 1 cm or less in its greatest dimension is considered as thyroid microcarcinoma ( 1 ). Papillary thyroid microcarcinoma is the most common form of thyroid microcarcinoma ( 2 ). It is associated with the risk of lymph node metastasis ( 1 , 3 ) and exhibits aggressive behavior ( 2 ). However, low-risk papillary microcarcinomas have excellent oncological outcomes of active surveillance ( 4 ). Papillary thyroid microcarcinoma is a subset of tumors with an indolent course and an even less aggressive treatment now proposed for these patients. However, some of these tumors may have higher rates of recurrent and persistent disease. Therefore, it is important to identify initial clinical and pathological characteristics that can predict a higher risk of progressive disease, avoiding undertreatment in this scenario. This is especially important for surgeons and clinical endocrinologists who face an increase in the incidence of papillary thyroid microcarcinoma today and must make accurate and economical treatment decisions in their routine.

Ultrasound imaging and fine-needle aspiration cytopathology are generally used for the diagnosis of papillary thyroid microcarcinoma ( 5 ). Ultrasound evaluates half of the lymph nodes due to the presence of thyroid ( 1 ). A retrospective chart review reported only 38% sensitivity of high-resolution ultrasound for predicting lymph node metastasis in papillary thyroid carcinoma ( 6 ). Japanese Society of Thyroid Surgeons (JSTS) ( 7 ) and Chinese Society of Clinical Oncology (CSCO) ( 8 ) guidelines suggested prophylactic lymph node resection to overcome complications regarding reoperations ( 8 , 9 ) but it is controversial in patients with papillary thyroid microcarcinoma ( 10 ) because of no evidence that rates of recurrence are decreased with this prophylactic lymph node resection ( 11 ). A retrospectively studies reported that lymph node metastasis frequency is higher in multifocal papillary thyroid microcarcinoma with higher sized nodules ( 1 , 5 , 10 ). The other retrospective study reported that clinicopathologic factors of papillary thyroid microcarcinoma of 5 or less mm diameter (Ø) nodules were less aggressive than more than 5 mm Ø nodules ( 2 ). A retrospectively studies reported that preoperative more than 65 pg/mL serum levels of calcitonin, subcapsular locations and the size of nodules are associated with lymph node metastasis ( 6 , 10 ). While retrospective studies reported that preoperative locations and the size of nodules are not associated with lymph node metastasis ( 2 , 5 ). Moreover, papillary thyroid microcarcinoma patients with clinically negative lymph node cancer have reported 3 % lymph node metastasis in the follow-up period after surgeries ( 12 ). Therefore, it is advisable to predict the association of clinicopathologic factors and preoperative sonographic features for papillary thyroid microcarcinoma for improvement of the diagnostic value of ultrasonography ( 2 ).

The aim of the retrospective study was to develop an association between clinicopathologic and sonographic features of patients with papillary thyroid microcarcinoma and the prevalence of lymph node metastasis.

## SUBJECTS AND METHODS

### Ethics approval and consent to participate

The first hospital of Lanzhou University, China approved the retrospective study (No. L-296) and waived the requirements of written informed consent form from the enrolled patients. Electronic medical records of patients have studied anonymously.

### Study population

**Figure 1:**
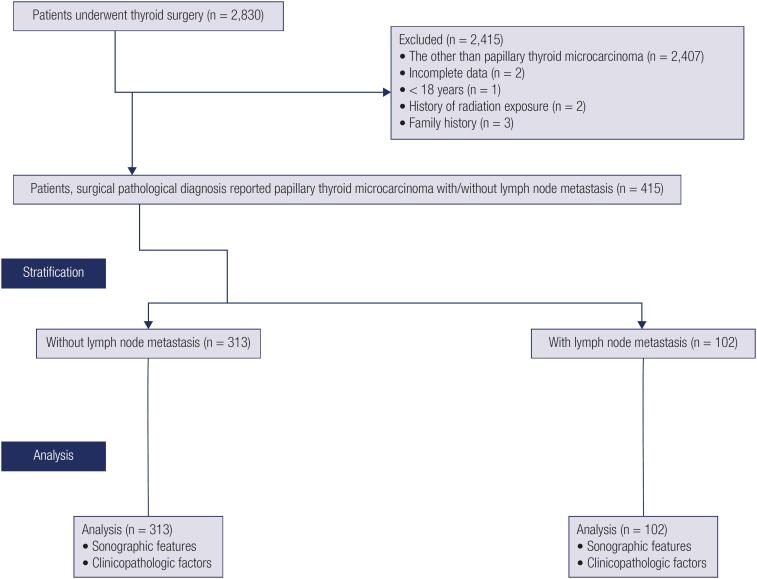
Retrospective study analysis chart.

From 15 January 2016 to 28 November 2019, a total of 2,830 patients underwent thyroid surgery (total thyroidectomy, lobectomy, or central compartment lymph node resection) at the first hospital of Lanzhou University, Lanzhou, China. Patients age ≥ 18 years, surgical pathological diagnosis reported papillary thyroid microcarcinoma (10 mm or less than in its maximum Ø) ( 9 ) with or without lymph node metastasis were retrospectively reviewed. 2,407 patients had other diagnosis than papillary thyroid microcarcinoma, two patients had incomplete data, one patient had age less than 18 years, two patients had a history of radiation exposure, three patients had a family history of thyroid carcinoma. Therefore, these patients were excluded from the study. In the case of multifocality, the largest nodule was used for analysis ( 5 ). Sonographic and clinicopathological data of 415 patients were used for analysis ( Figure 1 ).

### Ultrasonography

Grayscale and power Doppler ultrasound performed using ultrasound equipment (iU22, Philips Medical Systems, Amsterdam, Netherlands) with 7 MHz linear transducers. Cervical and thyroid sonography was performed in the longitudinal, transverse, and oblique planes. Ultrasound was performed by radiologists (minimum 3-years’ experience) of the institute. All ultrasound images were analyzed by ultrasound technologists (had 5-years of experience in thyroid imaging).

### Histopathology

It was performed for fresh surgically resected nodules of all patients by the pathologists (minimum 3-years’ experience) of the institute as per the 2004 World Health Organization (WHO) criteria. If two foci were found on at least one lobe it was considered bilaterality. Ø was calculated as the average of maximal diameter of all sides of nodules ( 1 ). The accompanying disease was considered Hashimoto's thyroiditis ( 2 ). Clinicopathologic parameters were collected by authors.

### Lymph node metastasis

After surgeries in follow-up, in grayscale or color Doppler ultrasound, the thickness of the lymph node ≥ 6 mm with intra-lymph nodal occupying lesions considered lymph node metastasis. Also, it was considered metastasis if lymph node perfusion or blood flow defect was found with any thickness size. If perfusion or blood flow defect found with any size thickness of the lymph node considered as metastasis ( 13 ). The suspected lymph nodes been submitted to fine needle aspiration for cytopathological confirmation. The decision of lymph node metastasis was reached by ultrasound technologists.

### Statistical analysis

SPSS v25.0 IBM Incorporation, Armonk, NY, United States was used for statistical analysis purposes. Constant data demonstrate frequency (percentage) and continuous data demonstrate mean ± SD. Fischer exact test for constant data and two-tailed unpaired *t* -test for continuous data performed for statistical analysis ( 1 ). Univariate following multivariate analysis was performed for the prediction of sonographic features and clinicopathologic factors for the prevalence of lymph node metastasis ( 3 ). All the results were considered significant at a 95% confidence level.

## RESULTS

### Sonographic features

A minimum of 1 nodule/patient and a maximum of 3 nodules/patients were reported. The other ultrasound parameters are reported in Table 1 . A total of 102 patients were developed lymph node metastasis in the follow-up time (the thickness of the lymph node ≥ 6 mm with intra-lymph nodal occupying lesions: 57 patients, lymph node perfusion: 37 patients, and blood flow defect: 8 patients).

**Table 1:** The preoperative ultrasound features of the enrolled patients

Characteristics		Population/value
Data of patients included in the analysis		415
Nodules/patient	Minimum	1
	Maximum	3
	Mean ± SD	1.21 ± 0.25
Distance to the carotid artery (mm)	Minimum	5.81
	Maximum	27.61
	Mean ± SD	11.92 ± 3.15
Depth	Minimum	4.11
	Maximum	23.12
	Mean ± SD	10.15 ± 4.15
Tumor capsular invasion	Yes	79 (19)
	No	336 (81)
Location of tumor	Right lobe	165 (40)
	Left lobe	90 (22)
	Isthmus	17 (4)
	Multicentric	143 (34)
Ratio of length/width	< 1	141 (34)
	≥ 1	274 (66)
Boundary	Clear	59 (14)
	Unclear	356 (86)
Peripheral halo ring		22 (5)
Hypoechogenicity		94 (23)
Isoechogenicity		21 (5)
Hyperechogenicity		5 (1)
Cystic change		29 (7)
Microcalcification		248 (60)
Macrocalcification		28 (7)
Normal vascularity		227 (55)
Hyper vascularity		61 (15)

Constant data demonstrate frequency (number) and continuous data demonstrate mean ± SD.

### Clinicopathologic factors

Among 415 patients 299 patients were female and 116 patients were male. The other demographical and clinicopathologic factors of patients are reported in Table 2 .

**Table 2:** The demographical and clinicopathologic factors of the enrolled patients

Characteristics		Population/value
Data of patients included in the analysis		415
Gender	Female	299 (72)
	Male	116 (28)
Age (years)	< 45	181 (44)
	≥ 45	234 (56)
	Mean ± SD	49.12 ± 8.47
Lymph node metastasis in follow up after surgery	Central lymph node metastasis	81 (20)
	Lateral lymph node metastasis	21 (5)
Body mass index (kg/m^2^)		24.81 ± 1.85
Ethnicity	Han Chinese	376 (91)
	Mongolian	34 (8)
	Tibetan	5 (1)
Preoperative calcitonin	≤65 pg/ mL	187 (45)
	>65 pg/ mL	228 (55)
Nodule size (mm; diameter)	≤5	176 (42)
	>5	239 (58)
	Mean ± SD	6.01± 0.35
Multifocality	Unilaterality	261 (63)
	Bilaterality	154 (37)
Hashimoto's thyroiditis		94 (23)
Follow-up time (months)		15.12 ± 8.12

Constant data demonstrate frequency (number) and continuous data demonstrate mean ± SD.

### Association of features for the prevalence of lymph node metastasis

Univariate analysis reported that tumor capsular invasion ( *p* < 0.0001), cystic change ( *p* = 0.001), and hypervascularity ( *p* = 0.015) of thyroid nodules were associated with lymph node metastasis ( Table 3 ).

**Table 3:** Univariate analysis for association of sonographic features for the prevalence of lymph node metastasis

Characteristics		No lymph node metastasis	Lymph node metastasis in follow up after surgery	Comparisons
Data of patients included in the analysis		313	102	*p-* value
Distance to the carotid artery (mm)	Minimum	5.81	6.01	0.054
	Maximum	27.61	25.12	
	Mean ± SD	11.12 ± 4.15	12.01 ± 3.69	
Depth	Minimum	4.11	5.01	0.298
	Maximum	22.99	23.12	
	Mean ± SD	10.03 ± 3.39	10.45 ± 4.01	
Tumor capsular invasion	* Yes	34 (11)	45 (44)	< 0.0001
	No	279 (89)	57 (56)	
Location of tumor	Right lobe	134 (43)	31 (30)	0.104
	Left lobe	68 (22)	22 (22)	
	Isthmus	12 (4)	5 (5)	
	Multicentric	99 (32)	44 (43)	
Ratio of length/width	< 1	108 (35)	33 (32)	0.719
	≥ 1	205 (65)	69 (68)	
Boundary	Clear	44 (14)	15 (15)	0.871
	Unclear	269 (86)	87 (85)	
Peripheral halo ring		16 (5)	6 (6)	0.798
Hypoechogenicity		72 (23)	22 (22)	0.892
Isoechogenicity		16 (5)	5 (5)	0.584
Hyperechogenicity		4 (1)	1 (1)	0.998
* Cystic change		14 (5)	15 (15)	0.001
Microcalcification		183 (59)	65 (64)	0.356
Macrocalcification		19 (6)	9 (9)	0.364
Normal vascularity		173 (55)	54 (53)	0.732
* Hyper vascularity		38 (12)	23 (23)	0.015

Constant data demonstrate frequency (number) and continuous data demonstrate mean ± SD. Fischer exact test for constant data and two-tailed unpaired *t* -test for continuous data performed for statistical analysis. A *p* < 0.05 considered significant.

* Significant parameter for lymph node metastasis.

Univariate analysis reported that male gender ( *p* < 0.0001), age < 45 years ( *p* = 0.011), and preoperative calcitonin level > 65 pg/ mL ( *p* < 0.0001), nodule Ø > 5 mm ( *p* < 0.0001), and multifocality of nodules ( *p* < 0.0001) were associated with lymph node metastasis ( Table 4 ).

**Table 4:** Univariate analysis for association of clinicopathologic factors for prevalence of lymph node metastasis

Characteristics		No lymph node metastasis	Lymph node metastasis in follow up after surgery	Comparisons
Data of patients included in the analysis		313	102	*p-* value
Gender	Male *	46 (15)	70 (69)	< 0.0001
	Female	267 (85)	32 (31)	
Age (years)	< 45 *	125 (40)	56 (55)	0.011
	≥ 45	188 (60)	46 (45)	
	Mean ± SD	54.12 ± 10.12	47.52 ± 7.12	
Body mass index (kg/m^2^)		25.02 ± 1.95	24.59 ± 2.11	0.059
Ethnicity	Han Chinese	286 (91)	90 (88)	0.562
	Mongolian	24 (8)	10 (10)	
	Tibetan	3 (1)	2 (2)	
Preoperative calcitonin	≤ 65 pg/mL	180 (58)	7 (7)	< 0.0001
	* > 65 pg/mL	133 (42)	95 (93)	
Nodule size (mm; diameter)	≤ 5	109 (35)	67 (66)	< 0.0001
	> 5 *	204 (65)	35 (34)	
	Mean ± SD	5.45 ± 0.25	7.15 ± 0.55	
Multifocality	Unilaterality	227	34	< 0.0001
	Bilaterality *	86	68	
Hashimoto's thyroiditis		69 (22)	25 (25)	0.589

Constant data demonstrate frequency (number) and continuous data demonstrate mean ± SD. Fischer exact test for constant data and two-tailed unpaired *t* -test for continuous data performed for statistical analysis. A *p* < 0.05 considered significant.

* Significant parameter for lymph node metastasis.

Multivariate analysis reported that male gender ( *p* = 0.041), age < 45 years ( *p* = 0.042), preoperative calcitonin > 65 pg/ mL ( *p* = 0.039), nodule size > 5 mm Ø ( *p* = 0.038), bilaterality ( *p* = 0.038), tumor capsular invasion ( *p* = 0.048), cystic change ( *p* = 0.047), and hyper vascularity ( *p* = 0.049) of thyroid nodules were associated with lymph node metastasis ( Table 5 ).

**Table 5:** Association of parameters with lymph node metastasis

Data of patients included in the analysis	102		
Parameters	Odd ratio	95 % confidence interval	*p-* value
Gender ( * male *vs.* female)	0.781	0.625–0.965	0.041
Age (< 45 * years *vs.* ≥ 45 years)	0.778	0.642–0.972	0.042
Preoperative calcitonin (> 65 * pg/ mL *vs.* ≤ 65 pg/mL)	0.762	0.632–0.985	0.039
Nodule size (> 5 * mm Ø *vs.* ≤ 5 mm Ø)	0.752	0.629–0.987	0.038
Multifocality ( * Bilaterality *vs.* Unilaterality)	0.743	0.665–0.998	0.038
Tumor capsular invasion ( * yes *vs.* no)	0.779	0.685–0.985	0.048
Cystic change ( * yes *vs.* no)	0.778	0.665–0.954	0.047
Vascularity ( * hyper vascularity *vs.* no vascularity)	0.776	0.663–0.952	0.049

Multivariate analysis. Data of absence lymph node metastasis considered the reference standard. A *p* < 0.05 considered significant.

* Significant parameter for lymph node metastasis.

Ø: Diameter.

## DISCUSSION

The study reported that tumor capsular invasion, cystic change, and hypervascularity of thyroid nodules were independent ultrasound features for the predictor of lymph node metastasis. The results of the study were agreed with the results of retrospective studies ( 2 , 3 , 6 , 10 ). Extra thyroidal extension of nodules ( 5 ) and multifocal lesions ( 1 ) have chances of lymph node metastasis. Calcification and vascularity increased as size increased ( 2 ). Preoperative ultrasound plays an important role in the prediction and management of lymph node metastasis in patients with papillary thyroid microcarcinoma.

The study reported that the male gender, age < 45 years, preoperative calcitonin > 65 pg/ mL, nodule size > 5 mm Ø, and bilaterality of nodules were independent clinicopathological parameters for the predictor of lymph node metastasis. The results of the study were agreed with the results of retrospective studies ( 1 – 3 , 5 , 9 , 10 ). Thyroid nodules > 5 mm Ø have high aggressiveness ( 2 ). Like radiological features, the clinicopathological characters also helpful for further thyroidectomy or radioiodine ablation.

The study used a 5 mm Ø threshold for the prediction of lymph node metastasis. The American Thyroid Association (ATA) guidelines recommended 5 mm Ø nodules for fine-needle aspiration biopsies ( 14 ). Papillary thyroid microcarcinoma changes little in size during a long follow-up ( 15 ). Therefore, the study used a 5 mm Ø nodule threshold to predict the aggressiveness of nodules for the prediction of lymph node metastasis.

The study recommended careful prophylactic surgery of lymph node in specific clinicopathological and sonographic features but has several limitations, for example, lack of a randomized case-control trial, separate parameters are required to evaluate for central and lateral lymph node metastasis. Follow-up data are not presented. The issue of generalizability because lymph node dissection is performed in China but not routinely performed in Western countries ( 1 ). A retrospective study reported that Tumor-node-metastasis (TNM) III and IV are associated with lymph node metastasis ( 9 ) but the current study did not evaluate the association of TNM staging for the association of lymph node metastasis. The possible justification is that it is not a surprising finding. As large numbers of surgeries and surgeons were involved. The degree of expertise also has effects on management of thyroidectomy. How many patients underwent lymph node dissection and what was rate of lymph node metastasis among these was not reported. Association between postoperative recurrence and lymph node metastasis did not report. The histopathological diagnosis of the tumor, classic, follicular, tall cell variant, and their an association with medullary carcinoma did not perform. All ultrasound images were not performed by ultrasound technologists but all ultrasound images were analyzed by ultrasound technologists. As being a retrospective study is known that histopathology was not performed by the same pathologist and it was not reviewed by a second or third pathologist.

In conclusion, besides the available guidelines, the retrospective study reported that the male gender, age < 45 years, preoperative calcitonin > 65 pg/mL, bilaterality, capsular invasion, cystic change, and hypervascularity of thyroid nodules associated with lymph node metastasis. Also, thyroid nodules 5 mm and more in diameter may have high aggressiveness. These data helped the surgeon for individualized treatment in thyroid carcinoma and avoid unnecessary prophylactic surgery of the lymph node. Chinese Society of Clinical Oncology diagnosis and treatment guidelines for thyroid cancer management is required to update.
